# Identification and genetic analysis of duck circovirus from selected regions of Jiangsu Province, China, in 2024

**DOI:** 10.3389/fvets.2026.1774530

**Published:** 2026-02-27

**Authors:** Yongxiao Tian, Xingdong Song, Xiaowei Wu, Mengxue Yuan, Yunwang Liu, Zijun Yang, Wenjie Wang, Shijin Jiang, Liangmeng Wei

**Affiliations:** 1 Sino-German Cooperative Research Centre for Zoonosis of Animal Origin of Shandong Province, Shandong Provincial Key Laboratory of Zoonoses, College of Veterinary Medicine, Shandong Agricultural University, Taian, China; 2 Guangzhou Customs Technology Center, Guangzhou, China; 3 Shuyang Zhongke Poultry Breeding Co., Ltd., Suqian, China

**Keywords:** duck circovirus, complete genome, genetic evolution, phylogenetic analysis, rep gene, Cap protein

## Introduction

Duck circovirus (DuCV) belongs to the genus *Circovirus* within the family *Circoviridae*. The genome of DuCV is a single-stranded circular DNA of approximately 2.0 kb that contains three major open reading frames (ORFs): ORF1, ORF2, and ORF3 (1–3). ORF1 encodes the replication protein (Rep), which is essential for viral replication (1). The protein possesses certain immunogenicity, and its amino acid sequence is highly conserved. ORF2 encodes the capsid protein (Cap), which is the main structural protein of the virus, possesses strong immunogenicity, and comprises the viral nucleocapsid. ORF3 is located on the complementary strand of ORF1 and possesses apoptotic activity (2). DuCV was first reported by Hattermann et al. (1) in Germany in 2003, and spread rapidly to Hungary (4), China (5), the United States (6), Poland (7), and South Korea (8). DuCV was first detected in mainland China in 2008 (5) and has been prevalent in many major duck-farming provinces, including Guangxi, Shandong, Anhui and Sichuan, causing significant economic losses to the duck industry. DuCV is divided into three major genotypes: DuCV-1, DuCV-2, and DuCV-3. DuCV-1 can be further divided into subtypes: DuCV-1a, DuCV-1b, DuCV-1c, and DuCV-1d, while DuCV-2 includes subtypes: DuCV-2a, DuCV-2b, and DuCV-2c (9, 10). DuCV-3 was first identified and isolated from domestic laying ducks in Hunan Province, China (11). DuCV infections often present with dry feathers, growth retardation, and respiratory distress. Pathological changes were observed in the liver, spleen, bursa of Fabricius, and thymus, and pathological examination revealed lymphocyte necrosis and histiocytic hyperplasia, which are closely associated with immunosuppression and lead to co-infections with duck hepatitis virus (DHV), duck enteritis virus (DEV), novel goose parvovirus (NGPV), and *Escherichia coli* (6, 12, 13), further increasing the mortality of infected duck flocks.

Jiangsu Province is one of the core waterfowl breeding provinces in Eastern China, adjacent to Shandong and other provinces with a high incidence of DuCV, resulting in a high risk of cross-regional viral transmission. However, there is currently a lack of systematic research on the epidemic genotypes, genetic evolution characteristics of strains, and protein mutation patterns of DuCV in Jiangsu Province. Therefore, this study conducted pathogen detection in suspected infected ducks in selected regions of Jiangsu Province, China. The complete genomes of positive samples were amplified and sequenced. Phylogenetic tree construction, sequence similarity analysis, and amino acid mutation site analysis were performed on the deduced amino acid sequences, aiming to clarify the epidemic and genetic evolution characteristics of DuCV in these selected regions, and provide a reference for the effective prevention and control of DuCV in Jiangsu Province.

## Materials and methods

### Viral sample collection

Between January and December 2024, 103 diseased duck tissue samples (including livers and spleens) from commercial duck flocks (e.g., Pekin duck, Muscovy duck) in three cities of Jiangsu Province (Suqian, Lianyungang, and Huai'an) were tested for pathogens in the laboratory. The samples identified as DuCV-positive were stored at – 80 °C for further use.

### Reference strains and dataset information

Genomic sequences of 47 DuCV reference strains were downloaded from the National Center for Biotechnology Information (NCBI) GenBank database, encompassing DuCV-1 and DuCV-2. Among these strains are prevalent isolates from China and a classic reference strain from the United States, to support a comprehensive analysis of genetic variations in the complete genome, *rep* gene, and *cap* gene. General information and accession numbers of these reference strains are provided in Supplementary Table 1.

### Primer synthesis and genome sequencing

The primers were designed according to previously published primer sequences for identification and full-length amplification (14) and were synthesized by Tsingke Biotechnology Co., Ltd., Qingdao, China. Total DNA was extracted from mixed liver and spleen tissue using a DNA extraction kit. Preliminary identification was performed through PCR using primers DuCV-F and DuCV-R. The amplification system consisted of 10 μl of 2 × Rapid Taq Master Mix, 2 μl of DNA template, 1 μl each of forward and reverse primers, and 6 μl of ddH_2_O, making a total volume of 20 μl. The amplification procedure entailed pre-denaturation at 95 °C for 3 min; 35 cycles of denaturation at 95 °C for 15 s, annealing at 55 °C for 15 s, and extension at 72 °C for 2 min. This was followed by a final extension at 72 °C for 5 min. The PCR products were analyzed by electrophoresis on a 1% agarose gel for identification. Positive samples identified by diagnostic primers were used as DNA templates for whole-genome amplification with primer pairs DuCV-F1/DuCV-R1, DuCV-F2/DuCV-R2, and DuCV-F3/DuCV-R3. The reaction system and program were the same as above, but the annealing temperatures were optimized to 53.4 °C, 52 °C, and 49.7 °C, respectively. Amplified products were detected by electrophoresis, purified by gel cutting, and sent to Sangon Biotech (Shanghai) Co., Ltd. for sequencing.

### Genetic evolutionary and sequence mutation analysis

The complete genome was processed using DNASTAR (Madison, WI, USA). Sequence alignment was performed using the database of the National Center for Biotechnology Information (NCBI, Bethesda, MD, USA). MEGA11 software (www.megasoftware.net) was used to construct a phylogenetic tree. DNASTAR was used to analyze nucleotide and amino acid homologies. BioEdit software was used to analyze amino acid mutation sites of the Rep and Cap proteins. Swiss-Model software was used to predict their three-dimensional structures, and PyMOL software was used to label the corresponding mutation sites.

### Ethics statement

The collection of duck tissue samples and subsequent experiments in this study were approved by Shandong Agricultural University Animal Care and Use Committee (Approval ID: SDAUA-2024-231). All procedures strictly adhered to relevant regulations. All tissue samples were obtained from ducks that died naturally or were culled due to disease on the farms and subsequently entered the harmless disposal process. Sample acquisition was conducted with the informed consent and permission of the source farms.

## Descriptive results

### DuCV detection and complete genome characterization

PCR identification results showed that nine out of 103 samples tested positive for DuCV. The nine DuCV strains were designated as DuCV/JS/1-2401/2024 (PX471094), DuCV/JS/2-2402/2024 (PX471095), DuCV/JS/3-2403/2024 (PX471096), DuCV/JS/4-2404/2024 (PX471097), DuCV/JS/5-2405/2024 (PX471098), DuCV/JS/6-2406/2024 (PX471099), DuCV/JS/7-2407/2024 (PX471100), DuCV/JS/8-2408/2024 (PX471101), and DuCV/JS/9-2409/2024 (PX471102), respectively (see Supplementary Table 2). Whole-genome amplification of these nine viruses yielded target fragments of 498, 1,211, and 478 bp. All nine strains were identified as DuCV genotype 1. Among them, DuCV/JS/8-2408/2024 was 1,995 nt in length, DuCV/JS/9-2409/2024 was 1,992 nt in length, and the other seven strains were all 1,993 nt in length.

### Complete genome homology and phylogenetic analysis

The complete genome homologies among the nine DuCV strains were 97.8%−99.8%, with the highest homology (99.8%) observed between DuCV/JS/1-2401/2024 and DuCV/JS/2-2402/2024. Compared with reference strains from Guangxi (e.g., DuCV-GX06-2019, DuCV-GX30-2020), Henan (e.g., AH02, HN02), Shandong (e.g., SDLC), Sichuan (e.g., DY01), and Taiwan (e.g., TC2/2002, TC3/2002), China, the nucleotide homology of the nine strains was 97.3%−99.6%, 97.7%−99.7%, 97.8%−99.5%, 98.1%−99.5%, and 82.0%−82.9%, respectively (see Supplementary Figure 1A). In addition, compared with representative strains of DuCV-1a subtype (e.g., Mulard duck/Germany/2003, Cherry Valley Pekin Duck/China/2009, Duck/Fujian/GQ423747/2008), the nucleotide homology of the nine strains was 94.1%−95.1%; while their nucleotide homology with representative strains of DuCV-1c subtype (e.g., LJ33, LJ07) was 92.1%−92.7%. The amino acid homology among the nine DuCV strains was 94.3%−99.6%, with the highest homology (99.6%) between DuCV/JS/1-2401/2024 and DuCV/JS/2-2402/2024. Compared with the reference strains from Guangxi (e.g., DuCV-GX54-2022, DuCV-GX30-2020), Henan (e.g., AH02, HN02), Shandong (e.g., SDLC), Sichuan (e.g., DY01), and Taiwan (e.g., Muscovy duck/China Taiwan/2002, TC3/2002), China, the amino acid homology of the nine DuCV strains with these reference strains was 93.1%−99.0%, 93.9%−99.4%, 94.8%−99.3%, 95.2%−99.1%, and 59.7%−61.9%, respectively. Additionally, the nine strains exhibited relatively high amino acid homology of 91.8%−94.0% with the classical DuCV-1b reference strain DQ100076 from the United States (see Supplementary Figure 2A).

**Figure 1:**
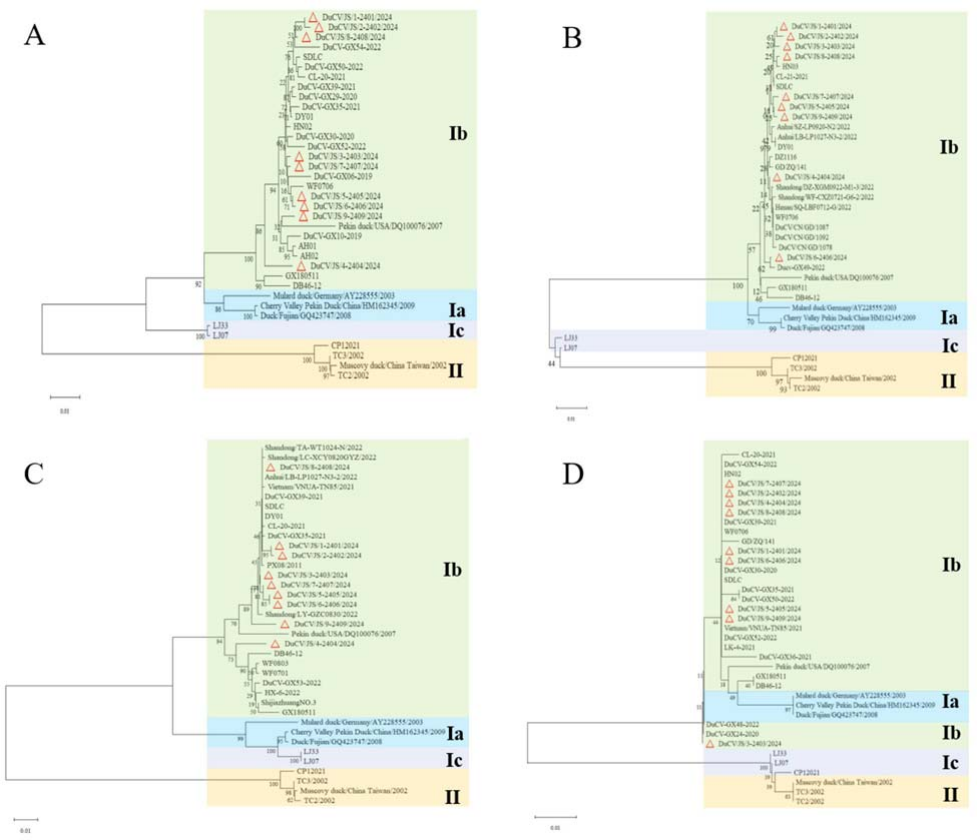
Phylogenetic trees of DuCV based on nucleotide sequences of the complete genome **(A)**, *rep* gene **(B)**, *cap* gene **(C)**, and *ORF3* gene **(D)**. The trees include sequences of nine DuCV strains from this study and sequences of reference strains retrieved from the GenBank sequence database (NCBI). Trees were reconstructed using the Neighbor-Joining (NJ) method with 1000 bootstrap replicates in MEGA 11 software.

Phylogenetic analysis based on the complete genome sequences revealed that all nine DuCV strains clustered in the same branch with the representative strains of the classical DuCV-1b subtype (e.g., DQ100076, GX180511, and DB46-12), but in distinct branches from the representative strains of the DuCV-1a subtype (e.g., Mulard duck/Germany/2003, Cherry Valley Pekin Duck/China/2009, and Duck/Fujian/GQ423747/2008), and DuCV-1c subtype (e.g., LJ33, LJ07), as well as the DuCV-2 reference strain from Taiwan, China. This indicates that these nine strains belong to the DuCV-1b genotype (Figure 1A).

### Rep gene homology and phylogenetic analysis

The *rep* gene nucleotide homology among the nine DuCV strains was 99.2%−99.9%, with the highest homology (99.9%) observed between DuCV/JS/5-2405/2024 and DuCV/JS/9-2409/2024. In addition, the nine DuCV strains had relatively high nucleotide homology with the reference strains from Guangxi (e.g., DuCV-GX49-2022), Guangdong (e.g., GD/ZQ/141, DuCV/CN/GD/1087, DuCV/CN/GD/1092), Henan (e.g., HN03, Henan/SQ-LBF0712-G/2022), Shandong (e.g., Shandong/DZ-XGM0922-M1-3/2022, SDLC), and Sichuan (e.g., DY01), but lower nucleotide homology with those from Taiwan (e.g., TC2/2002, TC3/2002), China (see Supplementary Figure 1B). Additionally, compared with representative strains of DuCV-1a subtype (e.g., Mulard duck/Germany/2003, Cherry Valley Pekin Duck/China/2009, Duck/Fujian/GQ423747/2008), the nine DuCV strains had a nucleotide homology of 97.8%−98.3%, with the highest homology (98.3%) observed between DuCV/JS/4-2404/2024 and Cherry Valley Pekin Duck/China/2009; their nucleotide homology with representative strains of DuCV-1c subtype (e.g., LJ33, LJ07) was 93.1%−93.3%. The amino acid homology among the nine DuCV strains was 99.7%−100%. The amino acid homology between the nine DuCV strains and the reference strains was high, although slightly lower than that among the nine strains themselves. Among these, DuCV/JS/9-2409/2024 and DuCV/CN/GD/1078 showed 99.7% amino acid homology, suggesting some genetic divergence (see Supplementary Figure 2B).

Phylogenetic analysis of the *rep* gene revealed that all nine DuCV strains clustered in the same branch with the representative strains of DuCV-1b genotype (e.g., DQ100076, GX180511, and DB46-12). Among them, DuCV/JS/1-2401/2024, DuCV/JS/2-2402/2024, DuCV/JS/3-2403/2024, and DuCV/JS/8-2408/2024 were closely related to the HN03 strain; DuCV/JS/6-2406/2024 was closely related to the DuCV-GX49-2022 strain; DuCV/JS/5-2405/2024, DuCV/JS/7-2407/2024, and DuCV/JS/9-2409/2024 were genetically closely related to each other; and DuCV/JS/4-2404/2024 was closely related to Henan/SQ-LBF0712-G/2022. Minor branch differences were observed compared with the phylogenetic tree of the *rep* gene (Figure 1B).

### Cap gene homology and phylogenetic analysis

The *cap* gene nucleotide homology among the nine DuCV strains was 96.0%−99.9%, with the highest homology (99.9%) observed between DuCV/JS/1-2401/2024 and DuCV/JS/2-2402/2024. Compared with the reference strains, DuCV/JS/4-2404/2024 had relatively high nucleotide homology with Shijiazhuang No.3, while the remaining eight strains had relatively high homology with DuCV-GX39-2021, SDLC, DY01, Anhui/LB-LP1027-N3-2/2022, and Shandong/TA-WT1024-N/2022. In addition, all nine DuCV strains had the lowest homology with TC2/2002 among all reference strains (see Supplementary Figure 1C). Additionally, compared with representative strains of DuCV-1a subtype (e.g., Mulard duck/Germany/2003, Cherry Valley Pekin Duck/China/2009, Duck/Fujian/GQ423747/2008), the nucleotide homology of the nine strains was 90.3%−91.6%; while their nucleotide homology with representative strains of DuCV-1c subtype (e.g., LJ33, LJ07) was 89.8%−91.1%. The amino acid homology among the nine DuCV strains was 96.5%−100%. DuCV/JS/3-2403/2024 and DuCV/JS/8-2408/2024 exhibited complete identity (100% homology), suggesting some differences compared with that of the nucleotide homology analysis. The nine DuCV strains had lower amino acid homology (78.4%−81.5%) with the four reference strains from Taiwan, China (see Supplementary Figure 2C).

Phylogenetic analysis of the *cap* gene revealed that all nine DuCV strains clustered in the same branch with the representative strains of DuCV-1b genotype (e.g., DQ100076, GX180511, and DB46-12). Among them, DuCV/JS/1-2401/2024, DuCV/JS/2-2402/2024, and DuCV/JS/8-2408/2024 were closely related to PX08/2011; DuCV/JS/3-2403/2024, DuCV/JS/5-2405/2024, DuCV/JS/6-2406/2024, and DuCV/JS/7-2407/2024 were genetically closely related to each other; DuCV/JS/4-2404/2024 was closely related to DB46-12, and DuCV/JS/9-2409/2024 was closely related to Shandong/LY-GZC0830. These results indicate that the nine DuCV strains exhibited genetic diversity in the *cap* gene (Figure 1C).

### ORF3 gene homology and phylogenetic analysis

The *ORF3* gene nucleotide homology among the nine DuCV strains was 99.6%−100%, indicating high similarity in the *ORF3* gene sequence. The homology between DuCV/JS/3-2403/2024 and each of the other eight strains was 99.6%, suggesting limited genetic diversity. In addition, the homology between the nine strains and other reference strains was also high (90.7%−100%) (see Supplementary Figure 1D). The amino acid homology among the nine DuCV strains was 98.7%−100%, further supporting the nucleotide homology analysis and indicating high conservation at the protein level. The homology between DuCV/JS/3-2403/2024 and the other eight strains was 98.7%, which was the lowest value among the nine strains. The nine DuCV strains had lower amino acid homology (78.5%−79.7%) with the four reference strains from Taiwan, China (see Supplementary Figure 2D).

Phylogenetic analysis of the *ORF3* gene revealed that all nine DuCV strains clustered in the same branch with the representative strains of DuCV-1b genotype (e.g., DQ100076, GX180511, and DB46-12). This was consistent with the complete genome phylogenetic tree. Among them, DuCV/JS/3-2403/2024 was closely related to DuCV-GX48-2022 and DuCV-GX24-2020, while the other eight DuCV strains were in the same branch (Figure 1D).

### Amino acid mutation analysis and protein structure prediction

Amino acid mutation analysis of the Rep protein in the nine DuCV strains revealed three mutations compared with the reference strain DQ100076. At position 122, strains DuCV/JS/1-2401/2024, DuCV/JS/2-2402/2024, DuCV/JS/3-2403/2024, and DuCV/JS/8-2408/2024 had an A→ G amino acid substitution. Additionally, all nine strains had a C→ G substitution at position 170 and an H→ L substitution at position 260 (see Supplementary Table 3). We further performed sequence alignment of the nine strains from this study with four representative Jiangsu DuCV strains (accession numbers: MF627687.1, MF627688.1, GU014543.1, OM867678.1). Results revealed that the C→ G substitution at position 170 and the H→ L substitution at position 260 were also present in the previously reported Jiangsu strains. However, the A→ G substitution at position 122 was unique to the four strains studied in this research.

For the Cap protein, amino acid mutation analysis showed that several of the nine strains had amino acid substitutions at positions 5 (T→ S), 23 (F→ L), 26 (R→ E), 35 (R→ L), 43 (K→ M), 194 (G→ S), 205 (K→ R), and 228 (K→ N) compared with DQ100076. In contrast, all nine strains had shared amino acid substitutions at positions 47 (N→ H/Y) and 56 (T→ Q) (see Supplementary Table 4). Further comparison with representative Jiangsu strains revealed that substitutions at positions 5, 47, 56, and 205 in the nine strains were also present in other Jiangsu strains. However, the F→ L substitution at position 23 was exclusively found in DuCV/JS/5-2405/2024, DuCV/JS/6-2406/2024, DuCV/JS/7-2407/2024, and DuCV/JS/9-2409/2024. The R→ E substitution at position 26 was unique to DuCV/JS/5-2405/2024 and DuCV/JS/6-2406/2024. The substitutions at position 35(R→ L), and position 43(K→ M), and position 228(K→ N) were all unique to DuCV/JS/4-2404/2024; the substitution at position 194(G→ S) was observed in both DuCV/JS/4-2404/2024 and DuCV/JS/9-2409/2024.

**Figure 2:**
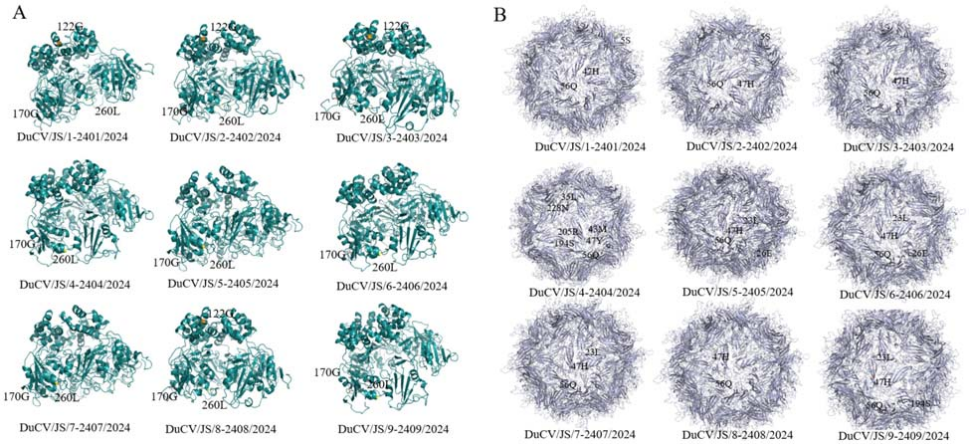
Predicted three-dimensional structures of DuCV Rep and Cap proteins. Rep **(A)** and Cap **(B)** protein structures predicted from nine DuCV strains.

The three-dimensional structures of the Rep and Cap proteins from the nine DuCV strains were predicted using the bioinformatics software Swiss-Model, with the further labeling of mutation sites performed via PyMOL. The results (Figure 2) showed that these protein structures exhibited high similarity in their overall conformations, consistent with their genetic relatedness. However, subtle differences were observed in specific local regions of both Rep and Cap proteins among different strains, indicating some genetic diversity.

## Conclusion

Comparative analysis of the complete genome sequences of the nine DuCV strains with the U.S. DuCV-1 reference strain DQ100076 and DuCV-1 strains from Southeastern China (HN02, WF0706) showed nucleotide identities of 97.7%−99.2%. Notably, DuCV/JS/5-2405/2024 and DuCV/JS/6-2406/2024 had a high genomic homology (99.4%) with the Shandong strain WF0706, suggesting that there may be a preliminary regional transmission chain of DuCV between the studied regions of Jiangsu Province and Shandong Province due to geographical adjacency and frequent circulation in the waterfowl industry. This conclusion is consistent with the findings of Wang et al. (15) that the DuCV-1d subtype strains in Anhui Province, China, have a close genetic relationship with Shandong strains due to the adjacency of the two provinces and frequent trade of duck products. Further identification in this study confirmed that all nine strains belonged to the DuCV-1b subtype, which is consistent with most previous studies in China, showing that DuCV-1b is the dominant subtype widely distributed in Northern and Southern China, further verifying the universal prevalence and geographical adaptability of this subtype in China. Furthermore, the monitoring results of DuCV in South Korea from 2013 to 2022 by Yu et al. (16) showed that the DuCV-1b subtype was the predominant strain in South Korea, indicating that DuCV-1b is the dominant subtype in global waterfowl breeding areas.

Among the nine DuCV strains, the nucleotide and amino acid homology of the Rep and ORF3 proteins was higher than that of the Cap protein. Compared with the reference strain DQ100076, the Rep protein exhibited fewer amino acid mutation sites than the Cap protein, demonstrating that the Cap protein is more prone to high-frequency mutations than the Rep protein. This result is consistent with the findings of Yu et al. (16) regarding the amino acid variation characteristics in South Korean DuCV sequences. Notably, as the major antigenic protein of DuCV, the amino acid differences and mutation sites in the Cap protein may influence its ability to induce neutralizing antibodies, and it is speculated that these mutations may assist the virus in evading host immune surveillance and clearance. The samples in this study covered selected duck-farming areas in Jiangsu Province, and the relevant conclusions need further verification by subsequent monitoring data with a wider coverage. However, this study clarified the genetic and evolutionary characteristics of prevalent strains in some regions of Jiangsu Province, supplemented the latest preliminary molecular epidemiological baseline data of DuCV in northern Jiangsu Province, provided an important foundation for further research on the genomic features of DuCV, and facilitated research on its epidemiology, pathogenic mechanisms, and control measures.

## Data Availability

The datasets presented in this study can be found in online repositories: GenBank accession numbers PX471094–PX471102.
