# Supplementary material for: Identification and genetic analysis of duck circovirus from selected regions of Jiangsu Province, China, in 2024
Source: Front Vet Sci. 2026 Feb 27;13:1774530. doi: 10.3389/fvets.2026.1774530 (PMC12982059; doi:10.3389/fvets.2026.1774530)
Supplement: Supplementary file 1 [file Data_Sheet_1.docx]

Supplementary Material

# Supplementary Figures and Tables

##
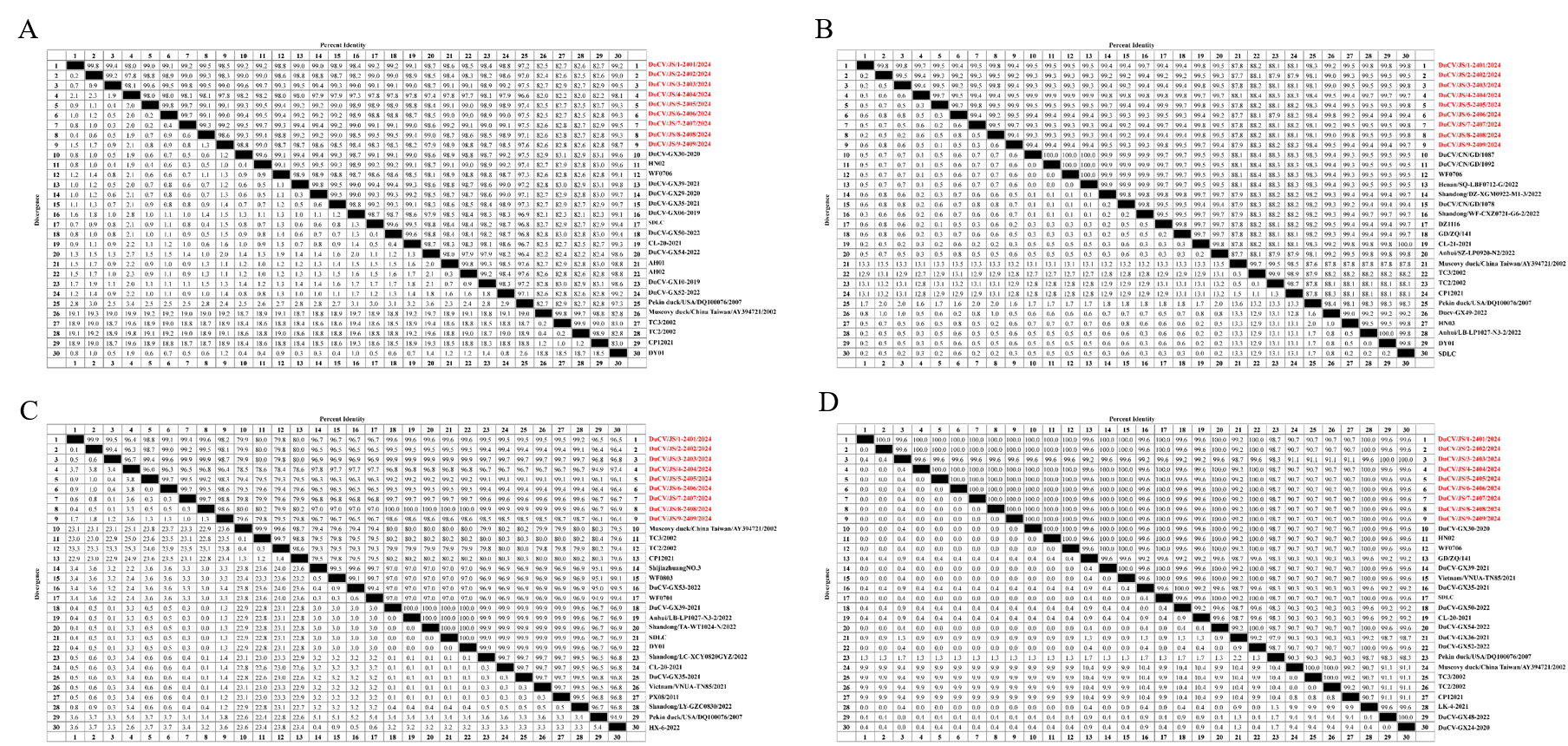
Supplementary Figures

**Supplementary Figure 1.** Comparison of nucleotide sequence homology between nine duck circovirus strains and reference strains A: Whole Genome Nucleotide Homology Analysis Results B: Rep Protein Nucleotide Homology Analysis Results C: Cap Protein Nucleotide Homology Analysis Results D: ORF3 Protein Nucleotide Homology Analysis Results

**
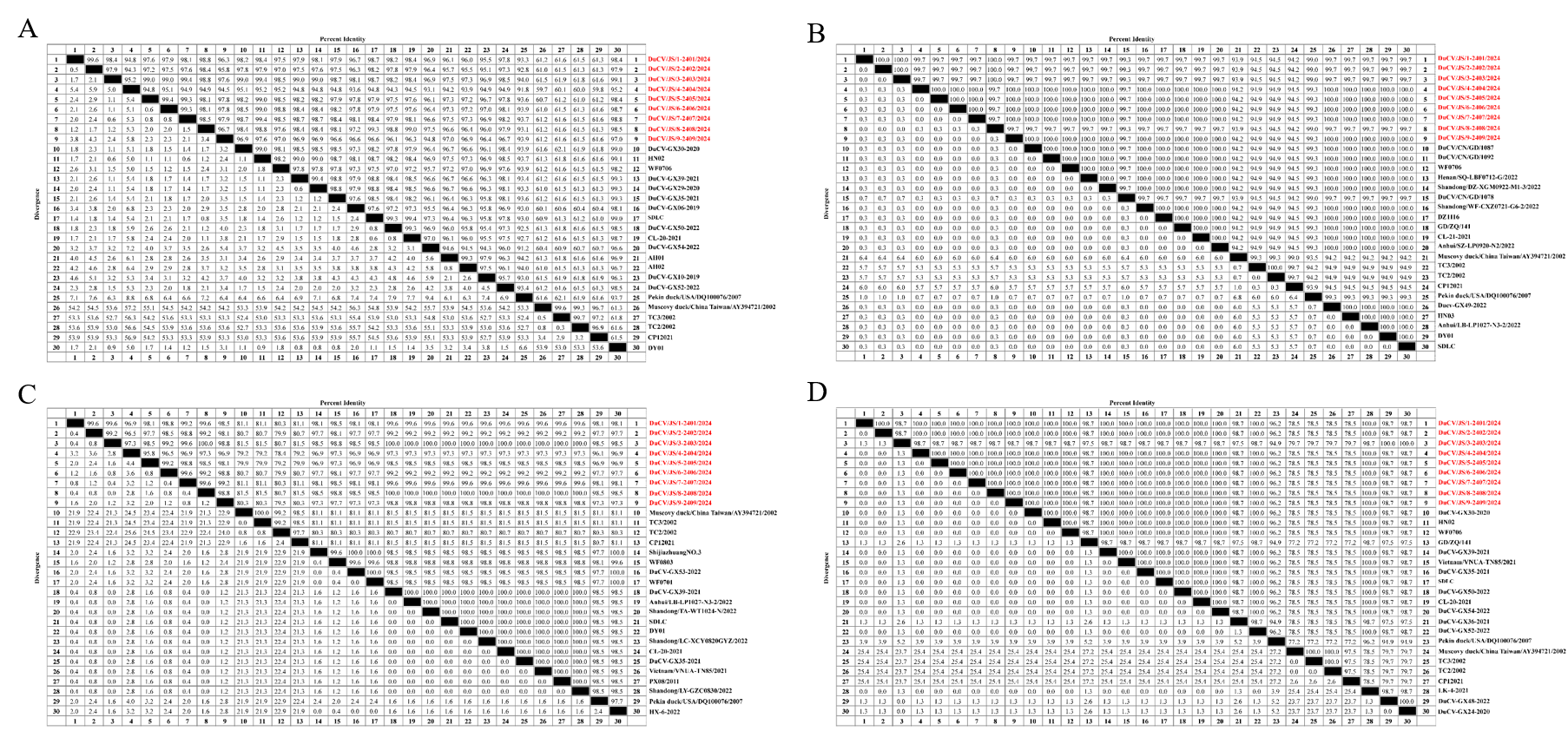
**

**Supplementary Figure 2.** Comparison of amino acid sequence homology between nine duck circovirus strains and reference strains A: Whole Genome Amino Acid Homology Analysis Results B: Rep Protein Amino Acid Homology Analysis Results
C: Cap Protein Amino Acid Homology Analysis Results D: ORF3 Protein Amino Acid Homology Analysis Results

## Supplementary Table

Supplementary Table1 Reference strains information

| Strains | GenBank  accession NO. | Years | Host | Area |
| --- | --- | --- | --- | --- |
| Du CV-GX30-2020 | OR134476.1 | 2020 | Duck | Nanning,Guangxi, China |
| HN02 | MN928795.1 | 2020 | Duck | Nanyang,Henan, China |
| WF0706 | OQ657183.1 | 2022 | Cherry Valley Duck | Tai’an,Shandong, China |
| Du CV-GX39-2021 | OR134510.1 | 2021 | Duck | Nanning,Guangxi, China |
| Du CV-GX29-2020 | OR134505.1 | 2020 | Duck | Nanning,Guangxi, China |
| Du CV-GX35-2021 | OR134508.1 | 2021 | Duck | Nanning,Guangxi, China |
| Du CV-GX06-2019 | OR134493.1 | 2019 | Duck | Nanning,Guangxi, China |
| SDLC | PP860902 | 2023 | Duck | Liaocheng,Shandong, China |
| Du CV-GX50-2022 | OR134513.1 | 2022 | Duck | Nanning,Guangxi, China |
| CL-20-2021 | ON756211.1 | 2021 | Duck | Zhengzhou,Henan, China |
| Du CV-GX54-2022 | OR134491.1 | 2022 | Duck | Nanning,Guangxi, China |
| AH01 | MN928808.1 | 2018 | Duck | Nanyang,Henan, China |
| AH02 | MN928809.1 | 2018 | Duck | Nanyang,Henan, China |
| Du CV-GX10-2019 | OR134466.1 | 2019 | Duck | Nanning,Guangxi, China |
| Du CV-GX52-2022 | OR134490.1 | 2022 | Duck | Nanning,Guangxi, China |
| Pekin duck/USA/DQ100076/2007 | DQ100076.1 | 2007 | Beijing Duck | The United States |
| Muscovy duck/ China Taiwan/AY394721/2002 | AY394721.1 | 2002 | Muscovy Duck | Taiwan, China |
| TC3/2002 | DQ166837.1 | 2002 | Muscovy Duck | Taiwan, China |
| TC2/2002 | DQ166836.1 | 2002 | Muscovy Duck | Taiwan, China |
| CP12021 | KP229377.1 | 2012 | Muscovy Duck | Taiwan, China |
| DY01 | OK094642.1 | 2020 | Duck | Chengdu,Sichuan, China |
| Du CV/CN/GD/1087 | OR842552.1 | 2023 | Duck | Foshan,Guangdong, China |
| Du CV/CN/GD/1092 | OR842553.1 | 2023 | Duck | Foshan,Guangdong, China |
| Henan/SQ-LBF0712-G/2022 | OR387752.1 | 2022 | Duck | Qingdao,Shandong, China |
| Shandong/DZ-XGM0922-M1-3/2022 | OR387760.1 | 2022 | Duck | Qingdao,Shandong, China |
| Du CV/CN/GD/1078 | OR842551.1 | 2023 | Duck | Foshan,Guangdong, China |
| Shandong/WF-CXZ0721-G6-2/2022 | OR387772.1 | 2022 | Duck | Qingdao,Shandong, China |
| DZ1116 | OQ657184.1 | 2022 | Cherry Valley Duck | Tai’an,Shandong, China |
| GD/ZQ/141 | ON227545.1 | 2021 | Duck | Foshan,Guangdong, China |
| CL-21-2021 | ON756217.1 | 2021 | Duck | Zhengzhou,Henan, China |
| Anhui/SZ-LP0920-N2/2022 | OR387727.1 | 2022 | Duck | Qingdao,Shandong, China |
| Du CV-GX49-2022 | OR134488.1 | 2022 | Duck | Nanning,Guangxi, China |
| HN03 | MN928796.1 | 2019 | Duck | Nanyang,Henan, China |
| Anhui/LB-LP1027-N3-2/2022 | OR387725.1 | 2022 | Duck | Qingdao,Shandong, China |
| ShijiazhuangNO.3 | MW255979.1 | 2019 | Duck | Baoding,Hebei, China |
| WF0803 | GU131342.1 | 2008 | Cherry Valley Duck | Tai’an,Shandong, China |
| Du CV-GX53-2022 | OR134514.1 | 2022 | Duck | Nanning,Guangxi, China |
| WF0701 | EU022375.1 | 2007 | Duck | Tai’an,Shandong, China |
| Shandong/TA-WT1024-N/2022 | OR387767.1 | 2022 | Duck | Qingdao,Shandong, China |
| Shandong/LC-XCY0820GYZ/2022 | OR387764.1 | 2022 | Duck | Qingdao,Shandong, China |
| Vietnam/VNUA-TN85/2021 | OM176555.1 | 2021 | Duck | Vietnam |
| PX08/2011 | KC460533.1 | 2011 | Duck | Nanning,Guangxi, China |
| Shandong/LY-GZC0830/2022 | OR387766.1 | 2022 | Duck | Qingdao,Shandong, China |
| HX-6-2022 | ON756215.1 | 2022 | Duck | Zhengzhou,Henan, China |
| Du CV-GX36-2021 | OR134509.1 | 2021 | Duck | Nanning,Guangxi, China |
| LK-4-2021 | ON756216.1 | 2021 | Duck | Zhengzhou,Henan, China |
| Du CV-GX48-2022 | OR134487.1 | 2022 | Duck | Nanning, Guangxi, China |
| Du CV-GX24-2020 | OR134471.1 | 2020 | Duck | Nanning, Guangxi, China |
| Mulard duck/Germany/2003 | AY228555.1 | 2003 | Duck | Germany |
| Cherry Valley Pekin Duck/China/2009 | HM162345.1 | 2009 | Cherry Valley Duck | Beijing, China |
| Duck/Fujian/GQ423747/2008 | GQ423747.1 | 2008 | Duck | Fujian, China |
| GX180511 | MK814578.1 | 2018 | Mulard duck | Guangxi, China |
| DB46-12 | KP229369.1 | 2013 | Pekin duck | Taiwan, China |
| LJ33 | EU344803.1 | 2008 | Mule duck | Fuzhou, Fujian, China |
| LJ07 | EU499311.1 | 2008 | Muscovy duck | Fuzhou, Fujian, China |

Supplementary Table 2 9 DuCV strain information

| Name of Isolate | Source | Age | Time |
| --- | --- | --- | --- |
| DuCV/JS/1-2401/2024 | Suqian, Jiangsu | 8w4 | 2024.7 |
| DuCV/JS/2-2402/2024 | Suqian, Jiangsu | 34w4/33w4 | 2024.7 |
| DuCV/JS/3-2403/2024 | Lianyungang, Jiangsu | 17d | 2024.7 |
| DuCV/JS/4-2404/2024 | Lianyungang, Jiangsu | 8w4 | 2024.7 |
| DuCV/JS/5-2405/2024 | Lianyungang, Jiangsu | 39d | 2024.8 |
| DuCV/JS/6-2406/2024 | Huai’an, Jiangsu | 15d | 2024.11 |
| DuCV/JS/7-2407/2024 | Suqian, Jiangsu | 26d | 2024.4 |
| DuCV/JS/8-2408/2024 | Lianyungang, Jiangsu | 35d | 2024.6 |
| DuCV/JS/9-2409/2024 | Suqian, Jiangsu | 17d | 2024.6 |

Supplementary Table 3 Comparison of Amino Acid Mutations in the Rep Protein Relative to the Reference Strain Pekin duck/USA/DQ100076/2007

| Designations of the Virus Strains | Amino acid position | | |
| --- | --- | --- | --- |
|  | 122 | 170 | 260 |
| DuCV/JS/1-2401/2024 | G | G | L |
| DuCV/JS/2-2402/2024 | G | G | L |
| DuCV/JS/3-2403/2024 | G | G | L |
| DuCV/JS/4-2404/2024 | A | G | L |
| DuCV/JS/5-2405/2024 | A | G | L |
| DuCV/JS/6-2406/2024 | A | G | L |
| DuCV/JS/7-2407/2024 | A | G | L |
| DuCV/JS/8-2408/2024 | G | G | L |
| DuCV/JS/9-2409/2024 | A | G | L |
| Pekin duck/USA/DQ100076/2007 | A | C | H |

## Supplementary Table 4 Comparison of Amino Acid Mutations in the Cap Protein Relative to the Reference Strain Pekin duck/USA/DQ100076/2007

| Designations of the Virus Strains | Amino acid position | | | | | | | | | | |
| --- | --- | --- | --- | --- | --- | --- | --- | --- | --- | --- | --- |
|  | 5 | 23 | 26 | 35 | 43 | 47 | 56 | 194 | 205 | 228 |  |
| DuCV/JS/1-2401/2024 | S | F | R | R | K | H | Q | G | K | K |  |
| DuCV/JS/2-2402/2024 | S | F | R | R | K | H | Q | G | K | K |  |
| DuCV/JS/3-2403/2024 | T | F | R | R | K | H | Q | G | K | K |  |
| DuCV/JS/4-2404/2024 | T | F | R | L | M | Y | Q | S | R | N |  |
| DuCV/JS/5-2405/2024 | T | L | E | R | K | H | Q | G | K | K |  |
| DuCV/JS/6-2406/2024 | T | L | E | R | K | H | Q | G | K | K |  |
| DuCV/JS/7-2407/2024 | T | L | R | R | K | H | Q | G | K | K |  |
| DuCV/JS/8-2408/2024 | T | F | R | R | K | H | Q | G | K | K |  |
| DuCV/JS/9-2409/2024 | T | L | R | R | K | H | Q | S | K | K |  |
| Pekin duck/USA/DQ100076/2007 | T | F | R | R | K | N | T | G | K | K |  |
